# SMA-Assisted Exfoliation of Graphite by Microfluidization for Efficient and Large-Scale Production of High-Quality Graphene

**DOI:** 10.3390/nano9121653

**Published:** 2019-11-21

**Authors:** Yuzhou Wang, Xianye Zhang, Haihui Liu, Xingxiang Zhang

**Affiliations:** 1 State Key Laboratory of Separation Membranes and Membrane Processes, Tianjin 300387, China; wangyuzhoumse@163.com (Y.W.); zhangxianye0630@163.com (X.Z.); haihui_liu@aliyun.com (H.L.); 2 Municipal Key Laboratory of Advanced Fiber and Energy Storage Technology, Tianjin 300387, China; 3 School of Material Science and Engineering, Tiangong University, Tianjin 300387, China

**Keywords:** liquid phase exfoliation, microfluidization, graphene, large-scale fabrication, polyamide 66 composites

## Abstract

In this paper, the sodium salt of styrene-maleic anhydride copolymer (SMA) was used as a stabilizer in the process of graphite exfoliation to few-layer graphene using the technique of microfluidization in water. This method is simple, scalable, and cost-effective, and it produces graphene at concentrations as high as 0.522 mg mL^−1^. The generated high-quality graphene consists of few-layer sheets with a uniform size of less than 1 μm. The obtained graphene was uniformly dispersed and tightly integrated into a polyamide 66 (PA66) matrix to create high-performance multifunctional polymer nanocomposites. The tensile strength and thermal conductivity of 0.3 and 0.5 wt% EG/PA66 composites were found to be ~32.6% and ~28.8% greater than the corresponding values calculated for pure PA66, respectively. This confirms that the new protocol of liquid phase exfoliation of graphite has excellent potential for use in the industrial-scale production of high-quality graphene for numerous applications.

## 1. Introduction

Graphene is a novel two-dimensional carbon material that is of great interest because of its unprecedented properties, including high Young’s modulus (~1 TPa), great strength (130 GPa) [1], large specific surface area (~2630 m^2^ g^−1^) [2], and good thermal conductivity (5000 W m^−1^ K^−1^) [3]. The combination of such remarkable characteristics in graphene renders this material useful in many applications, such as field-effect transistors, supercapacitors, batteries, sensors, and nanocomposites [4,5,6,7]. Consequently, it is crucial to develop efficient methods that allow for the production of pure graphene in high yields. Typically, graphene may be prepared via chemical vapor deposition, epitaxial growth, mechanical exfoliation, or chemical oxidation [8,9,10,11], with the latter being the most common method used to produce the material on a large scale. However, the graphene generated by chemical oxidation has many defects, and the process usually requires exigent conditions and leads to pollution.

Recently, liquid phase exfoliation (LPE) of graphite powder to few-layer graphene has attracted the interest of many researchers [12,13,14]. This novel technique was first proposed by Coleman et al. for the production of graphene via sonication in solvents [15]. Since then, surfactants and polymers have been incorporated into the solvent to assist in the exfoliation process [16,17]. However, LPE methods are not suitable for large-scale applications, as they are characterized by high energy consumption and low exfoliation efficiency [18]. Contrarily, microfluidization is considered as an effective method for the synthesis of few-layer graphene on a large scale, because of its low energy consumption and availability. This homogenization technique is based on the use of high pressure to force a fluid through a narrow channel. It relies on three simultaneous effects (cavitation, collision, and high shear stress) to peel graphene material off from the bulk graphite [18].

To enhance the yield and quality of the graphene produced by LPE or microfluidization methods, stabilizers, such as aqueous surfactants and polymers, are used. However, most of the currently used stabilizers, such as sodium dodecyl benzene sulfonate (SDBS) [19], sodium cholate (SC) [16], polyvinyl pyrrolidone (PVP) [17], and sodium dodecyl sulfate (SDS) [20], are characterized by low graphene production yields (maximum concentration of 0.3 mg mL^−1^). This shows that more effective low-cost stabilizers need to be developed for use in large-scale graphene preparation procedures.

Styrene-maleic anhydride (SMA) copolymer is a polymer dispersant that is widely used in the fields of biology, medicine, optics, electronics, among others [21]. The functional sodium salt derivative of this copolymer is a low-cost, green, and hydrophilic compound that comprises a lot of benzene rings in its chemical structure and thus may be used to promote π–π interactions between graphene layers. Despite the great potential of SMA in the direct exfoliation of graphite, investigations of the stabilizing activity of this compound are scarce.

This study constitutes the first reported investigation of the efficiency of SMA in facilitating graphite exfoliation via microfluidization in an aqueous system. The effects of SMA concentration, microfluidization cycle number, and chamber pressure on the effectiveness of graphene production are systematically analyzed, and an exfoliation mechanism is proposed. The exfoliated graphene is used to prepare graphene/polyamide 66 (PA66) composites and graphene films by melt blending injection and vacuum filtration, respectively, and the mechanical, thermal, and electrical properties of these materials are analyzed.

## 2. Materials and Methods

### 2.1. Materials

Pristine graphite (medium particle size of 5–10 μm, carbon purity of 95%) was provided by Qingdao Tengshengda Carbon Machinery Co., Ltd., in China. Meanwhile, the emulsion of SMA sodium salt (SMA, 2.5 wt%) and the polyamide 66 (EPR27) pellets were purchased from Shanghai Leather Chemical Factory and Pingdingshan Shenma Co., Ltd., in China, respectively. SDBS was provided by Shanghai Aladding Chemical Reagent Co., Ltd., China.

### 2.2. Exfoliation of Graphite to Graphene

**Figure 1:**
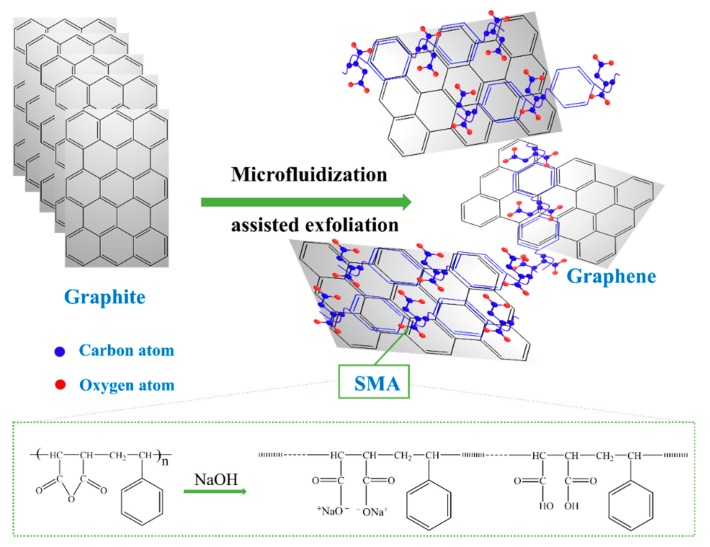
Illustration of graphite exfoliation to graphene using the technique of styrene-maleic anhydride (SMA)-assisted microfluidization.

The SMA stabilizer was added to 10 mg mL^−1^ graphite solutions at concentrations of 5, 10, and 15 mg mL^−1^. The 500 mL SMA/graphite mixtures were stirred for 30 min to ensure homogeneity, then they were exfoliated using a lab-scale pressure homogenized microfluidizer (M-110L, Microfluidics, USA). The chamber pressure was varied between 80 and 120 MPa, and the number of cycles completed through the interaction chamber (cycle number) was set to 5, 10, 20, or 30. Upon the termination of the microfluidization process, the graphite suspensions were centrifuged at 3000 rpm for 30 min to remove un-exfoliated graphite sheets, and the topmost part (80%) was collected. Then, the exfoliated graphene (EG) dispersions were subsequently vacuum filtrated through a 0.22-μm pore size membrane, and then freeze dried to obtain EG powder. The chemical transformations implicated in graphite exfoliation by SMA-assisted microfluidization in water are illustrated in Figure 1. For comparison, the conventional surfactant SDBS was also used to assist the microfluidization exfoliation process.

Microfluidization is a homogenization technique that uses high pressure to pass fluids through a narrow channel (<100 μm). Typically, graphite exfoliation is achieved using either normal or lateral mechanical forces. In microfluidization, the two mechanisms are combined to attain high exfoliation efficiency. The process is characterized by three main phenomena: cavitation, shear, and collision [22]. Cavitation occurs when the fluids pass through the narrow channel, resulting in the generation of the normal force needed for exfoliation. The high pressure applied to the flow leads to collision and turbulence, which induces a shear stress force. The velocity gradient generated by the sudden release of pressure at the end of the channel also produces viscous shear stress. The combination of generated stress promotes the self-exfoliation of graphite down to single or few-layer graphene by lateral self-lubrication [18].

**Figure 2:**
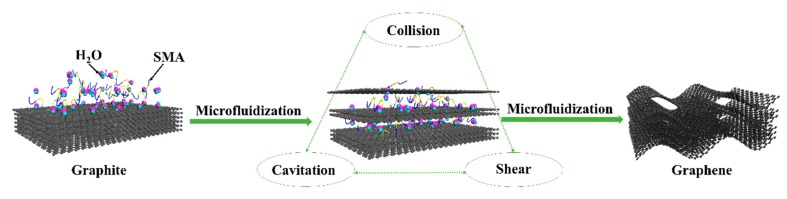
Scheme of the SMA-assisted liquid phase exfoliation method used to produce graphene from graphite.

SMA assists in the exfoliation process by incorporating itself between the carbon sheets in such a way that it minimizes the interactions between them. The stabilizer is composed of long chain molecules that bind to the surface of graphene sheets via non-covalent π–π interactions. The interacted SMA forms a coating layer with repulsive tails that prevent interactions with other graphene sheets. The process of SMA-assisted liquid phase exfoliation of graphite is illustrated in Figure 2.

### 2.3. Fabrication of Graphene/PA66 Composites

The exfoliated graphene powder was dispersed in commercially bought PA66 pellets by mechanical mixing for 3 h. The mixtures containing 0.1, 0.2, 0.3, 0.4, and 0.5 wt% EG were subsequently fed into a twin-screw extruder (Hartec, HTDG-16, Lanxi, China) operated at a melting temperature of around 280 °C and rotation speed of 100 rpm. The extrudates were pelletized, dried, and then molded to tensile specimens using an injection molding machine (SZS-20, Wuhan Ruiming, Wuhan, China).

### 2.4. Characterization

The graphene dispersion was analyzed by UV-visible spectroscopy (Agilent Cary 60, Carpinteria, CA, Australia) in the range of 200–800 nm, using a quartz cell with an optical path of 1 cm. All dispersions were diluted 10 times for UV-Vis analysis. The morphology of as-prepared graphene was observed by transmission electron microscopy (TEM, Hitachi H-800, Tokyo, Japan) at 100 kV, whereas the thickness of the material was determined using atomic force microscopy (AFM, CSPM55OO, Guangzhou, China) in tapping mode. The structures of SMA, graphite, and graphene were evaluated using a Raman spectrometer (XploRA PLUS, Tokyo, Japan) equipped with a 532 nm excitation laser, as well as a Fourier-transform infrared (FTIR) spectrometer (NICOLET 6700, Waltham, MA, USA) scanning in the range of 4000–400 cm^−1^. Meanwhile, the crystallinity of the investigated material was analyzed using an X-ray diffractometer (D8 DISCOVER, Karlsruhe, Germany) operated in the range of 10–60° (2*θ*) and/or a differential scanning calorimeter (DSC, NETZSCH 200 F3, Selb, Germany) scanning between −30 °C and 300 °C at the rate of 10 °C min^−1^ under a nitrogen atmosphere. The surface morphologies and mechanical properties of pure PA66 and EG/PA66 composites were analyzed using a field-emission scanning electron microscope (SEM, Hitachi S4800, Tokyo, Japan) and a universal tensile machine (CMT-4203, Xinsansi, Shanghai, China), respectively. For strength testing, dried dumbbell specimens were prepared, and their mechanical properties were evaluated at a tensile speed of 50 mm min^−1^. The tests were repeated five times, and only the average values are reported. The thermal conductivities of pure PA66 and its EG-modified composites were also assessed at 25 °C using an analyzer (Netzsch LFA 447 Nanoflash, Selb, Germany).

## 3. Results and Discussion

### 3.1. Optimization of the Exfoliation Process

The concentration of graphene in the prepared EG dispersions (*C*_G_) was calculated using the Lambert–Beer law [19]

**Figure 3:**
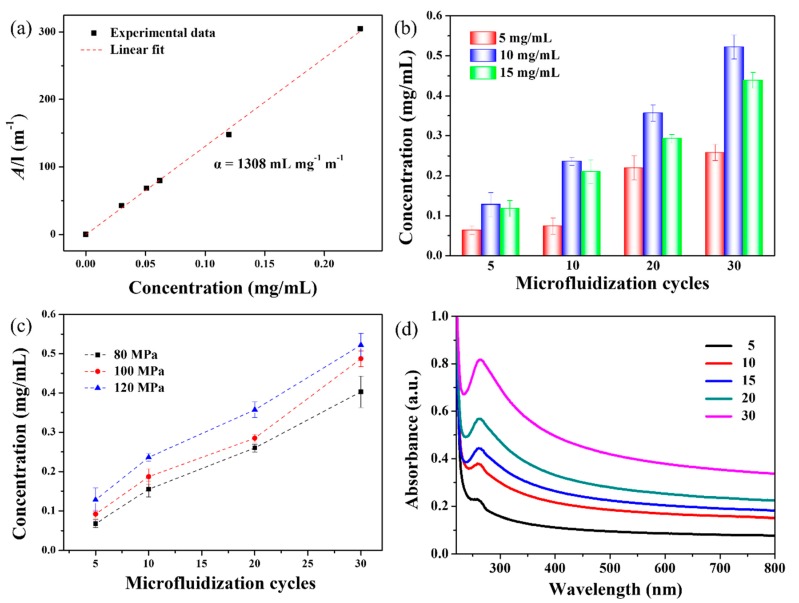
Correlation between UV-visible spectroscopy (UV-Vis) absorbance and graphene concentration (**a**). Influence of the number of microfluidization cycles on graphene concentration in EG dispersions prepared using different amounts of SMA (**b**), and different pressures (**c**). UV-vis spectra of EG dispersions (**d**).

*A* = *α*·*C*·*l*, (1)

where *A* is the absorbance value at 660 nm, measured using UV-Vis spectroscopy, *α* is the absorption coefficient, *C* is the concentration of graphene, and *l* is the optical path length (*l* = 1 cm). The value of *α* was determined based on the absorbance of graphene dispersions whose concentrations (*C*_G_) are known. For this purpose, samples of EG suspensions were analyzed by UV-Vis spectroscopy then vacuum filtrated through a 0.22-μm pore size membrane. The collected solid was thoroughly washed by water to eliminate excess SMA, then it was dried and weighed to determine the mass of graphene, which was used to calculate *C*_G_. Finally, *α* was estimated based on the slope of the linear fit between *A* and *C*_G_. As shown in Figure 3a, the calculated value of *α* for the SMA-stabilized graphene dispersions prepared in this study is 1308 mL·mg^−1^·m^−1^. Henceforth, the concentrations of EG dispersions are determined using this value.

The influence of SMA concentration (5, 10, and 15 mg·mL^−1^) on *C*_G_ was assessed under a microfluidization pressure of 120 MPa. The results presented in Figure 3b demonstrate that maximum values of *C*_G_ are attained at *C*_SMA_ = 10 mg·mL^−1^, irrespective of the number of microfluidization cycles. A higher concentration of SMA will not promote further increase in *C*_G_ because of the graphene surface that becomes saturated with adsorbed SMA molecules [23].

The effects of pressure and number of cycles are also analyzed, as shown in Figure 3c. The results indicate that an increase in either one of these two factors results in a higher concentration of graphene. During microfluidization, graphene is peeled off from the graphite by the effects of collision, shear force, and the energy generated from cavitation [18]. At higher pressures, more energy is produced, which facilitates the exfoliation of graphene. In addition, the increase in the number of microfluidization cycles enhances the absorption of cavitation shock waves by graphite, which leads to reduced interaction forces between the layers of this material and ultimately facilitates exfoliation [22]. In this study, the maximum value of *C*_G_ (0.522 mg·mL^−1^) is recorded at 120 MPa and 30 cycles. Pressures beyond 120 MPa were not investigated, because the pressure is adjusted by the gap valve. In fact, pressures higher than this value result in agglomeration and blockage during the microfluidization process. To compare the exfoliation effect between SMA and the conventional surfactant, SDBS was also used to assisted the microfluidization exfoliation. Under the same condition of microfluidization process (*C*_SDBS_ = 10 mg·mL^−1^, 120 MPa, 30 cycles), the resulted graphene concentration assisted by SDBS was as low as 0.328 mg·mL^−1^, which indicates that SMA is a relatively efficient stabilizer for the synthesis of graphene.

The UV-Vis spectra used to calculate *C*_G_ offer additional evidence of the successful exfoliation of graphite to graphene. As shown in Figure 3d, all SMA-stabilized dispersions are characterized by flat spectra with a single peak at ~270 nm. This peak is characteristic of the π–π* transition of C–C bonds in graphene [24], and thus, it is used to confirm the successful exfoliation of this product [24]. Consistent with the *C*_G_ data, greater absorbance is observed at higher numbers of microfluidization cycles, indicating an improved exfoliation effect. The UV-Vis spectra recorded in this study are similar to those reported by Phiri [24] and Pu et al. [25].

### 3.2. Characterization of As-Prepared Graphene

**Figure 4:**
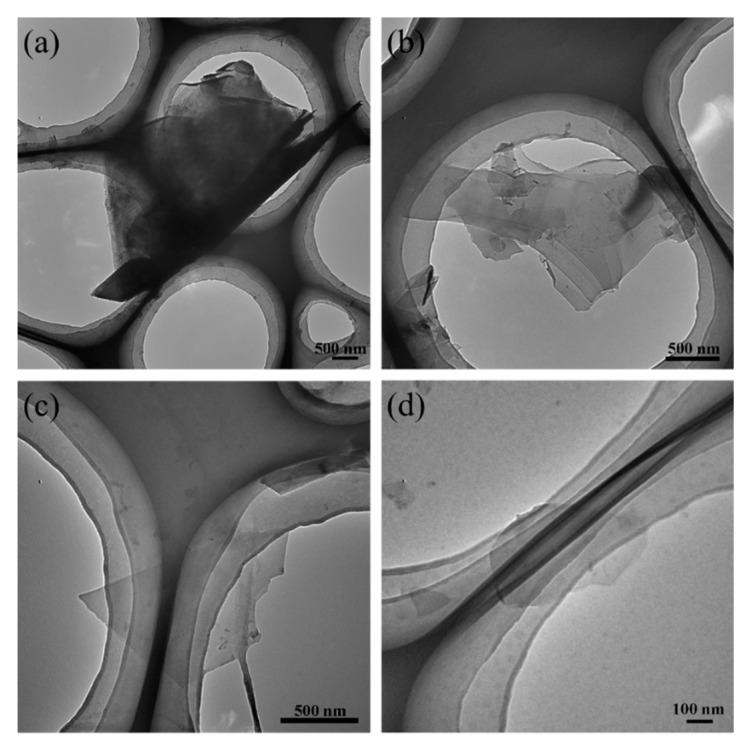
Transmission electron microscopy (TEM) micrographs of the graphene prepared using 5 (**a**), 10 (**b**), 20 (**c**), and 30 (**d**) microfluidization cycles.

The effect of the number of microfluidization cycles on graphene structure was analyzed using TEM (Figure 4). Figure 4a shows that, after five cycles of microfluidization, a very thick structure is produced. Meanwhile, the graphene collected after ten cycles has a multilayer structure, compared to the few- and single-layer structures observed after 20 and 30 cycles, respectively. In addition, the size of graphene decreases with increasing number of cycles, which suggests that more microfluidization cycles promote better exfoliation.

**Figure 5:**
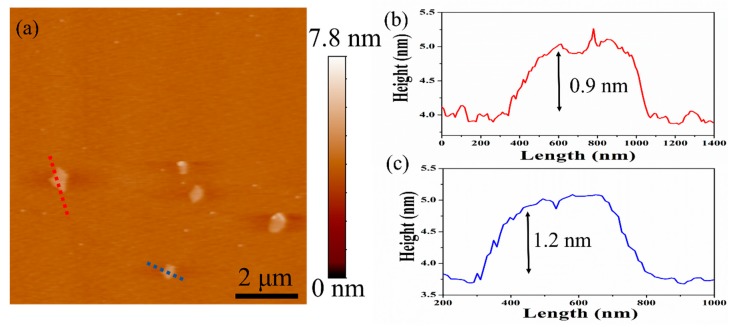
Atomic force microscopy (AFM) micrograph (**a**) and cartograms (**b**,**c**) of selected graphene sheets produced after 30 cycles of microfluidization.

The thickness of the graphene produced after 30 cycles of microfluidization was measured by AFM (Figure 5a), and the height profiles of selected graphene sheets were determined. According to the data presented in Figure 5b,c, the thickness of as-prepared graphene sheets is about 0.9 and 1.2 nm. These values are appreciably greater than the theoretical thickness of monolayer graphene (0.334 nm) because of the attached SMA molecules on the surface and the folded structure of the material. Actually, similar observations are reported in other AFM studies [12,23,26]. Therefore, it could be concluded that the graphene produced by SMA-assisted microfluidization is mainly a few-layer (less than 5) structure with a lateral size of 0.5–0.9 μm, which is consistent with the TEM results.

**Figure 6:**
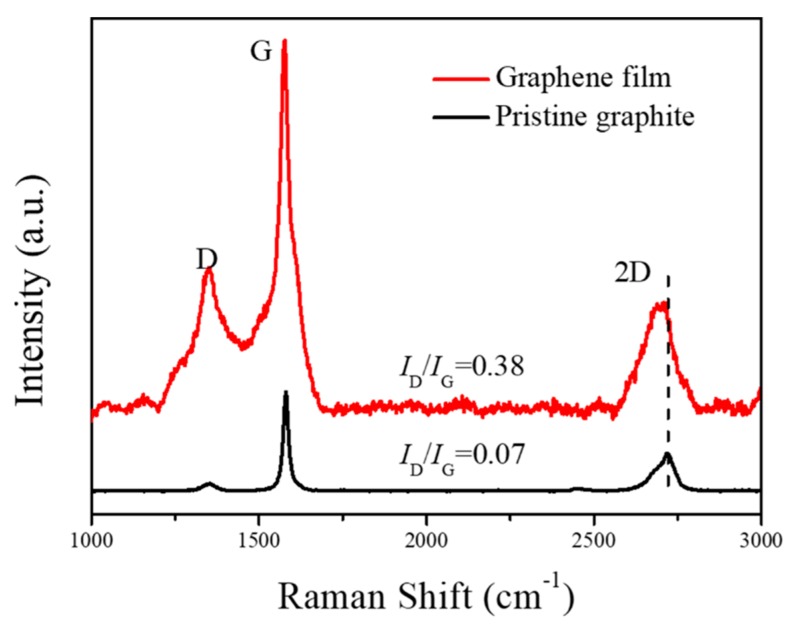
Raman spectra of pristine graphite and the as-prepared graphene film.

The Raman spectrum of the prepared graphene was recorded and compared to that of graphite after 30 cycles of microfluidization under 120 MPa. The data illustrated in Figure 6 show three main peaks for graphene at around 1350 (D), 1580 (G), and 2700 cm^−1^ (2D). The degree of defect in EG is determined based on the ratio of *I*_D_/*I*_G_; a lower ratio signifies a higher quality of the graphene flake. Based on the Raman spectrum depicted in Figure 6, the *I*_D_/*I*_G_ ratio increases from 0.07 for pristine graphite to 0.38 for as-prepared graphene because of the structural defects generated during the exfoliation process. Nevertheless, the *I*_D_/*I*_G_ ratio obtained in this study using SMA-assisted microfluidization (0.38) is comparable with those achieved using other LPE methods (0.2–0.5) [14,27,28], which suggests that the quality of the graphene prepared herein is relatively high.

**Figure 7:**
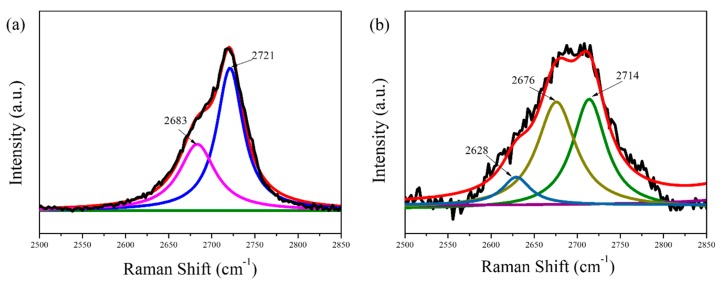
Enlarged images with fitting curves of 2D-band of pristine graphite (**a**) and the as-prepared graphene film (**b**).

The 2D-band of pristine graphite and the as-prepared graphene film was fitted using the non-linear least square fit program (Gauss–Lorentzian). After deconvolution, the objective results with high correlation are shown in Figure 7. The 2D-band of the pristine graphite exhibited two-peaks profile (2683 and 2721 cm^−1^), typical to graphite. By contrast, three fitting curves are clearly observed in the spectra of the graphene products, as shown in Figure 7b. The first peak (2628 cm^−1^) belongs to the monolayer or bilayer graphene sheets. However, the second peak (2676 cm^−1^) and the third peak (2714 cm^−1^) are attributed to few-layer and multilayer graphene sheets in products [29]. The exfoliation of monolayer graphene from graphite was not achieved which may be due to the re-aggregation of graphene layers during the film formation. In addition, compared to graphite, the 2D band in graphene is shifted to lower wavenumbers, which suggests that the material consists of less than five layers [30]. Furthermore, the 2D band becomes more symmetrical after exfoliation, thereby confirming the presence of few-layer graphene [29].

**Figure 8:**
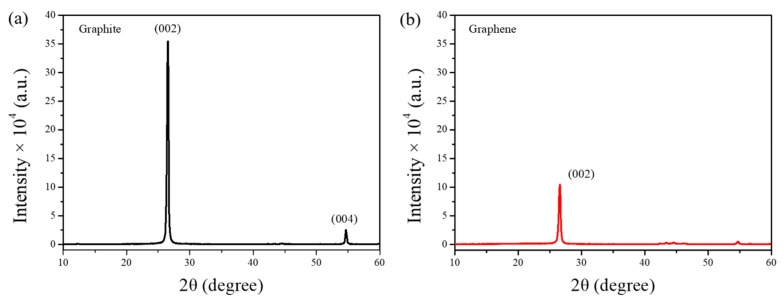
X-ray diffraction (XRD) spectra of graphite (**a**) and as-prepared graphene (**b**).

The crystallization of EG was analyzed using XRD and compared to the crystal formation in pristine graphite. As shown in Figure 8, the XRD pattern of graphite presents two intense peaks at 26.7° and 54.4°, corresponding to the (002) and (004) crystal planes, respectively. Meanwhile, the spectrum of EG shows a single low-intensity peak attributed to the (002) plane and a negligible (004) peak, which further confirms the exfoliation of graphite to few-layer graphene [13]. The residual diffraction detected at 26.7° may be ascribed to the re-aggregation and re-stacking of graphene flakes during water evaporation [26].

**Figure 9:**
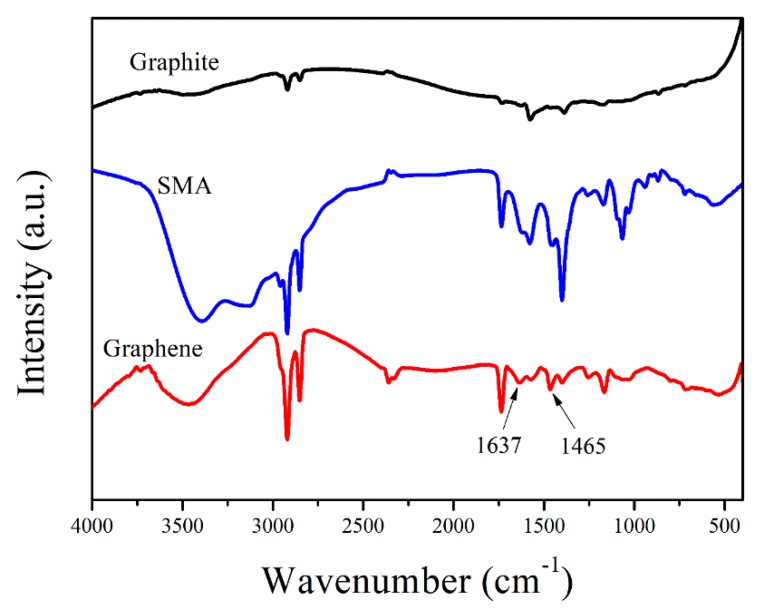
Fourier-transform infrared (FTIR) spectra of graphite, SMA, and EG.

FTIR spectra of graphite, SMA, and graphene confirm the existence of π–π interactions between the stabilizer and the carbon sheets. As shown in Figure 9, the spectra of EG and SMA are relatively similar. However, the characteristic peaks corresponding to aromatic ring breathing vibrations at 1625 and 1451 cm^−1^ are significantly shifted (to 1637 and 1465 cm^−1^, respectively) in the EG sample. This suggests that the IR peaks observed in the graphene spectrum are mainly due to SMA molecules that are non-covalently bound to the material via π–π interactions [13].

**Figure 10:**
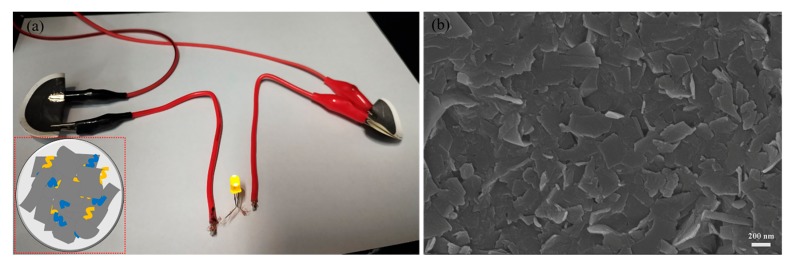
Photograph showing the setup used for electrical conductivity testing of graphene thin films (the inset illustrates the gluing of graphene sheets by SMA molecules at inter-flake junctions) (**a**). SEM micrograph of the graphene film (**b**).

The electrical conductivity of graphene films prepared by vacuum filtration was measured using the four-point probe technique. Before testing, the films were dried overnight in a vacuum oven set at 50 °C. As shown in Figure 10a, the graphene films prepared in this study may conduct enough electricity to light up a 3 V LED lamp. The SEM micrograph (Figure 10b) of the graphene film indicates that the size of the material belongs to the sub-micrometer scale, which is consistent with the results of TEM and AFM analyses. In addition, unlike graphite, graphene sheets are randomly stacked in parallel alignment, leading to enhanced electronic properties of the material, particularly conductivity [31]. In fact, the electrical conductivity value of the prepared graphene is about 7500 S m^−1^, which is greater than the values determined for the same material produced by sonication-based LPE methods (6000–6500 S m^−1^) [15,31].

### 3.3. Application of As-Prepared Graphene in Polyamide 66 Composites

The results presented thus far prove that high-quality graphene may be generated in relatively large concentrations via SMA-assisted microfluidization in water. This green approach is characterized by high efficiency and low cost, which renders it useful for the fabrication of graphene-based polymer composites, particularly when compared to the alternative CVD and GO (graphene oxide) reduction methods. Polyamide 66 (PA66) is an engineering plastic that is widely used because of its good processability and excellent mechanical properties [32]. However, these properties still do not meet the exacting demands of highly specialized industries, such as aerospace and automobile industries. In this study, we assessed the effect of the prepared graphene on the mechanical properties of PA66. The presence of SMA stabilizer on the graphene surface is expected to facilitate the dispersion of the material in the PA66 matrix, resulting in strong interface compatibility between the two entities. Moreover, the uniform sub-micrometer size of EG should enhance the reinforcement of PA66 composites [33,34].

**Figure 11:**
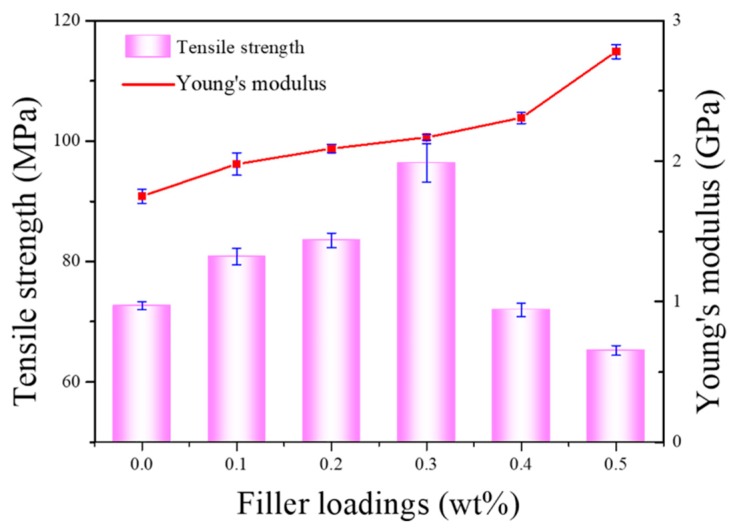
The effect of filler loading on the tensile strength and Young’s modulus of pure PA66 and EG/PA66 composites.

The effect of EG content on the mechanical properties of PA66 is illustrated in Figure 11. Based on the obtained results, the tensile strength of PA66 composites increases with increasing EG content up to 0.3 wt%. Beyond this value, tensile strength decreases, probably because of the aggregation of graphene [33]. The maximum strength attained is 96.36 MPa, which is 32.6% greater than the value recorded for pure PA66. Similarly, Young’s modulus increases with increasing EG load up to 0.5 wt%. This is consistent with the results published in the literature [35]. Compared to other graphene-reinforced polyamide composites [32,36,37], as shown in Table 1, greater improvements in mechanical properties are observed for the EG/PA66 composites investigated in this study.

**Figure 12:**
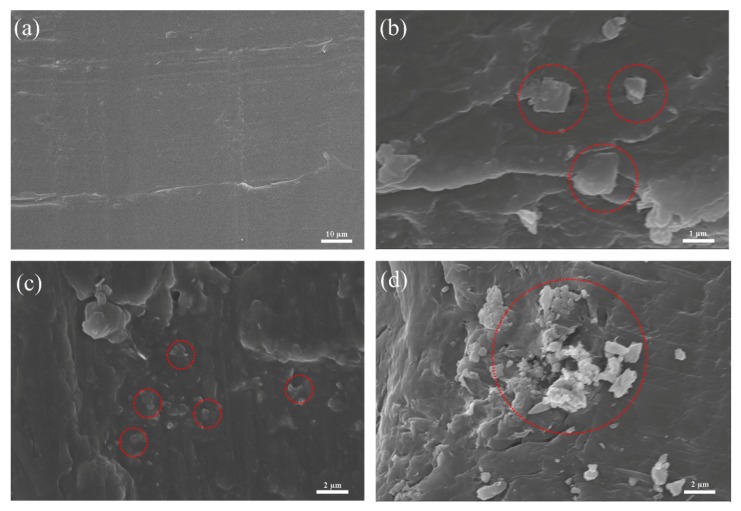
Scanning electron microscopy (SEM) micrographs of pure PA66 (**a**), 0.1% EG/PA66 (**b**), 0.3% EG/PA66 (**c**), and 0.5% EG/PA66 (**d**) fracture surfaces.

It is well-known that the mechanical properties of composites are enhanced by good dispersibility and strong interfacial adhesion between the modifier and the matrix. Therefore, we used SEM to characterize these properties in the EG/PA66 composites. The SEM micrographs in Figure 12 show pieces of graphene dispersed on the flat and smooth fracture surface of PA66. When graphene loading is less than 0.3 wt%, the modifier uniformly disperses in the matrix, leading to well-combined composite material with strong interface interactions. Meanwhile, when the loading is over 0.3 wt%, graphene sheets become aggregated because of the high surface area of nanoplates and strong π–π interactions between layers (Figure 12d). These properties result in the concentration of stress, which significantly reduces tensile strength [33,35,36].

**Figure 13:**
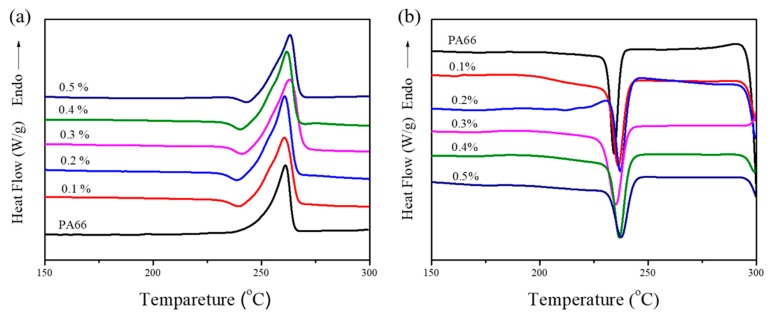
Differential scanning calorimeter (DSC) curves of the melting (**a**) and cooling (**b**) behavior in pure PA66 and EG/PA66 composites.

In addition to dispersibility, crystallinity is another factor that influences the mechanical properties of EG/PA66 composites [33]. In this work, DSC tests were performed to investigate the crystal formation behavior in pure PA66 and EG/PA66 composites. The DSC curves and data presented in Figure 13 and Table 2, respectively, show that the melting temperatures (*T*_m_) of the investigated composites are practically the same, irrespective of the EG content. This indicates that the samples possess analogous lamellae thicknesses and spherulite sizes [37]. Moreover, the EG/PA66 composites exhibit higher crystallization temperatures (*T*_c_) compared to pure PA66, which signifies that EG acts as an effective heterogeneous nucleating agent that promotes crystallization [33,37]. The greatest crystallinity (*X*_c_ = 36.6%) is achieved using 0.3 wt% EG, leading to the highest tensile strength of the composites. Excessive EG (>0.3 wt%) results in unavoidable reaggregation, which may impede the movement of molecular chains, thereby reducing the crystallinity [38]. These results are highly consistent with the mechanical properties of pure PA66 and EG/PA66 composites.

**Figure 14:**
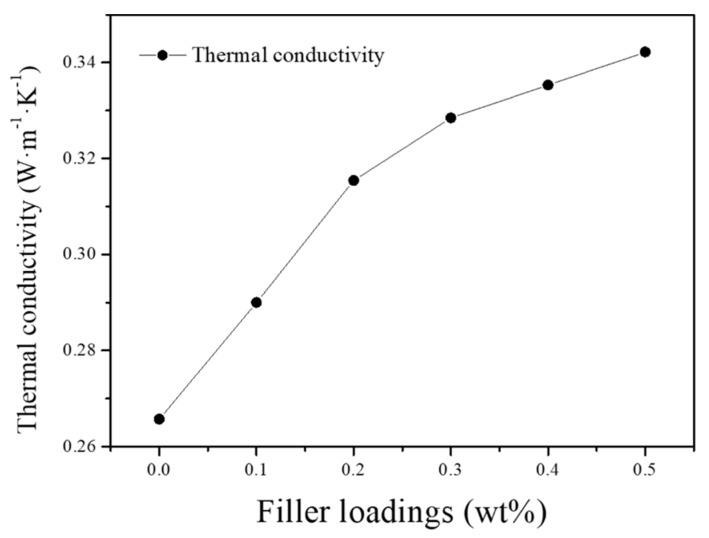
Thermal conductivity of PA66 and EG/PA66 composites.

Figure 14 shows that the thermal conductivity of the fabricated EG/PA66 composites increases consistently with increasing EG content. In fact, the conductivity of the 0.5 wt% EG/PA66 composite (0.3422 W·m^−1^·K^−1^) is 28.8% greater than that of the pure PA66 (0.2657 W·m^−1^·K^−1^). Previously, it has been shown that the incorporation of 0.25 wt% graphene in silicone foams increases thermal conductivity by only 6% [39]. Therefore, the SMA-stabilized graphene produced herein is much more efficient in enhancing the thermal conductivity of composite material than alternative graphene materials. The demonstrated improvement is attributed to the strong interaction between EG and PA66 matrix, which is induced by the transfer of thermal energy in the form of phonons. Moreover, the coupling of vibrational modes at the EG-PA66 interface reduces the generation of significant thermal resistance [40], leading to enhanced thermal conductivity. Thus, the new method of EG production is useful in the preparation of EG-based polymers with improved properties. These materials have great potential for use in a wide range of applications, such as connectors and other high-performance thermal management systems.

## 4. Conclusions

In summary, we report a new and green method for the exfoliation of graphite to few-layer graphene, based on the technique of SMA-assisted microfluidization in water. This method yields graphene concentrations as high as 0.522 mg mL^−1^ and a uniform lateral size of less than 1 μm. Compared to graphene oxide and reduced graphene oxide, the EG synthesized in this study is of high quality (*I*_D_/*I*_G_ = 0.38) and has good electrical conductivity (7500 S m^−1^). Furthermore, the process of exfoliation is fast and waste-free, which renders it suitable for large-scale production. When combined with polymers such as PA66, SMA-stabilized EG shows good dispersion and interface compatibility, which significantly reinforces the EG/PA66 composites. Overall, this study presents a green and facile approach to produce high-quality graphene and paves the way for the fabrication of high-performance multi-functional graphene/polymer composites.

## Figures and Tables

**Table 1:** Comparison of literature data with this work for the mechanical properties of PA66 composites.

Filler	Loading (%)	Tensile Strength (MPa)	Young’s Modulus (GPa)	Reference
Exfoliated graphene plates	1	77.7	3.09	[32]
Acid-treated carbon nanotubes	10	40.0	1.40	[36]
Exfoliated graphene plates	0.5	90.5	2.85	[37]
EG	0.3	96.36	2.17	This work

**Table 2:** DSC data of pure PA66 and EG/PA66 composites.

Filler Loading (wt%)	*T*_m_ (°C)	Δ*H*_m_ (J/g)	*T*_c_ (°C)	Δ*H*_c_ (J/g)	*X*_c_ (%)
0	261.1	48.04	234.0	46.75	24.9
0.1	260.6	59.06	237.2	58.4	31.1
0.2	260.7	65.72	237.2	67.29	35.8
0.3	263.1	67.34	235.0	68.72	36.6
0.4	261.8	58.63	236.7	55.50	29.5
0.5	263.2	52.46	237.6	55.23	29.4
